# Vein of Galen malformation in newborns and children: contemporary advances and future directions

**DOI:** 10.3389/fped.2026.1996440

**Published:** 2026-10-16

**Authors:** Silvia Buratti, Andrea Rossi, Darren B. Orbach

**Affiliations:** 1 Neonatal and Pediatric Intensive Care Unit, Emergency Department, IRCCS Istituto Giannina Gaslini, Genoa, Italy; 2 Department of Neuroscience (DINOGMI), University of Genoa, Genoa, Italy; 3 Department of Health Sciences (DISSAL), University of Genoa, Genoa, Italy; 4 Neuroradiology Unit, Services Department, IRCCS Istituto Giannina Gaslini, Genoa, Italy; 5 Neurointerventional Radiology, Boston Children’s Hospital, Harvard Medical School, Boston, MA, United States

**Keywords:** cerebrovascular malformations, endovascular procedures, fetal therapies, genetics, neurodevelopmental outcomes, neuroimaging, Vein of Galen malformations

## Abstract

**Background:**

Vein of Galen malformation is a rare and complex cerebrovascular disorder associated with significant morbidity and mortality from the fetal period through childhood. Advances in perinatal care, neuroimaging, endovascular therapy, and multidisciplinary management have improved survival and expanded therapeutic options, while generating new challenges in clinical strategies and long-term outcomes.

**Objective:**

This review integrates evidence and expert perspectives presented during the II International Meeting on Vein of Galen Malformation in Newborns and Children, held in Camogli, Italy, in October 2025 and summarizes current advances, emerging developments, and priorities for research and clinical innovation.

**Recent advances:**

Current developments include improved fetal and neonatal imaging, emerging prognostic biomarkers, fetal intervention, advances in endovascular treatment, and increasing understanding of underlying genetic and molecular mechanisms. Contemporary management increasingly integrates cardiovascular and neurological assessment, advanced neuroimaging, individualized endovascular strategies, and structured long-term neurodevelopmental follow-up. The development of a disease-specific registry reflects the growing impact of international collaboration.

**Future directions:**

Major priorities include validation of risk-stratification tools, development of standardized imaging and neurodevelopmental biomarkers, refinement of fetal and neonatal interventions, improved understanding of disease biology, and multicenter data sharing. Family-centered care and standardized long-term outcome assessment should remain integral components of future VOGM research and clinical practice.

**Conclusions:**

VOGM management is rapidly evolving from survival-focused treatment toward individualized, multidisciplinary care extending from fetal diagnosis through childhood and adulthood. This review provides a comprehensive overview of current knowledge and emerging developments in this field, while defining a multidisciplinary, internationally informed roadmap for future research.

## 1 Introduction

The II International Meeting on Vein of Galen Malformation in Newborns and Children, held in Camogli, Italy, in October 2025, brought together experts from fetal medicine, neonatology, pediatric neurology, neuroradiology, neurointerventional radiology, neurosurgery, genetics, and allied disciplines. Building upon the momentum of the inaugural meeting in 2023, this second gathering further consolidated an expanding international network committed to improving the understanding, diagnosis, and treatment of VOGM.

VOGM remains a challenging condition due to its rarity, heterogeneity, and the intricate interplay between genetic, cerebrovascular, cardiac, and developmental factors. Over recent decades, advances in neuroimaging, neonatal intensive care, and endovascular techniques have significantly improved survival. However, these improvements have also revealed new challenges, particularly concerning the biological basis of the disease, optimal therapeutic strategies, and long-term neurodevelopmental outcomes. Throughout all sessions, a central theme emerged, the necessity of multidisciplinary collaboration and international data sharing to address unanswered questions and improve patient outcomes.

In this review, we synthesize these developments and place them in the context of the current literature, with particular emphasis on prenatal diagnosis, risk stratification, fetal and neonatal intervention, neuroimaging, disease biology, long-term outcomes, and international collaboration.

## 2 Historical perspectives and conceptual foundations

The historical evolution of knowledge surrounding arteriovenous fistulas, particularly VOGM, provides essential context for the development of contemporary diagnostic and therapeutic strategies for this complex condition.

Early observations of vascular anomalies date back to the 18th century, but it was not until the 20th century that the distinct nature of VOGM began to be recognized. The first published case of VOGM dates to a detailed and illustrated article by Jaeger in 1946 (1). Contributions from pioneers in neurosurgery and neuroradiology laid the groundwork for understanding the embryological origin of the malformation, particularly the role of the median prosencephalic vein of Markowski. The first successful treatment of VOGM dates back to 1949 and involved experimental ligation of the carotid artery (2). However, a full understanding of the anomaly's developmental basis was only achieved thanks to several important publications by Raybaud and colleagues (3–5). Notably, Raybaud's realization that the lesion incorporates the median prosencephalic vein of Markowski confirmed its choroidal nature and timing of formation.

A critical turning point in the field was the shift from surgical to endovascular treatment (6). The introduction of embolization techniques in the late 20th century revolutionized management, enabling targeted, minimally invasive interventions. Contemporary approaches now include transarterial, transvenous, hybrid, and even fetal techniques, reflecting the rapid evolution of the field.

Importantly, historical insights highlighted that VOGM is not a static entity but part of a broader spectrum of arteriovenous shunts. This recognition has significant implications for classification, diagnosis, and individualized treatment planning.

## 3 Perinatal management and fetal intervention

A major focus of recent research has been the perinatal period, where early diagnosis and risk stratification are critical determinants of outcome. Advances in prenatal imaging—particularly ultrasound and fetal magnetic resonance imaging (MRI)—have enabled earlier detection of VOGM and more detailed assessment of associated cardiovascular and neurological involvement. Despite these advances, predicting perinatal clinical courses remains challenging. Multiple prenatal markers—such as cardiomegaly, tricuspid regurgitation, and abnormal flow patterns—have been proposed, but no single parameter reliably predicts prognosis (7). In this regard, prenatal neuroradiological factors—such as straight-sinus or falcine-sinus mediolateral diameter or the presence of pseudofeeders—represent potential markers for high-risk neonatal presentation and worse outcomes (8–10). A particularly important topic is the identification of this high-risk subgroup of newborns who require early neonatal intervention, often within the first week of life. These patients tend to have significantly poorer outcomes, highlighting the need for highly specialized and innovative approaches (7–9). Multiparametric models integrating cardiovascular, anatomical, and neurological data have been proposed as a promising tool for stratifying post-natal clinical courses (7).

One of the most important recent developments is the emergence of fetal embolization as a potential therapeutic strategy (11, 12). Early experience demonstrates technical feasibility and substantial reduction in cardiac output. Single-center experience demonstrates technical feasibility and suggests the potential for improved outcomes in carefully selected high-risk fetuses; however, the small sample size, absence of a concurrent control group, short follow-up, and substantial risk of unscheduled delivery preclude conclusions regarding efficacy (11, 12).

Complementary preclinical work in animal models demonstrated the feasibility of transcardiac fetal arterial access for embolization (13), opening a new potential approach for prenatal therapy. These findings suggest that fetal intervention may represent an additional paradigm shift in the management of severe VOGM.

## 4 Cardiovascular pathophysiology and neonatal management

VOGM imposes a significant haemodynamic burden on the fetus and newborn due to a large intracranial arteriovenous shunt that increases cardiac preload and can lead to high-output heart failure. During fetal development, the low-resistance and high-capacitance uteroplacental circulation exerts a protective role for the fetus. However, after birth, the sudden loss of placental circulation and lowering of pulmonary vasculature resistance result in dramatic hemodynamic imbalance, including severe pulmonary overflow, systemic steal, and rapid progression to cardiac failure. Neonatal management has evolved toward a pathophysiology-driven approach (7, 14, 15). The primary goals include stabilizing cardiac function, optimizing systemic perfusion, and preparing the patient for endovascular intervention. Strategies involve tailored ventilation, targeted use of inotropic and vasoactive agents, and avoidance of therapies that may exacerbate pulmonary overcirculation (e.g., pulmonary vasodilators, such as inhaled nitric oxide). Ductal patency active management (via prostaglandin E1) has been specifically reported for patients with refractory pulmonary hypertension (7). These physiological considerations support early multidisciplinary assessment and individualized hemodynamic management, highlighting the synergistic roles of intensivists and cardiologists in close coordination with neurointerventional teams to optimize timely treatment, survival, and long-term outcomes in high-risk newborns.

## 5 Advances in neuroimaging

Neuroimaging plays a pivotal role in all stages of VOGM management, from prenatal diagnosis to treatment planning and follow-up. The meeting highlighted the transition from purely anatomical imaging to a more comprehensive, multiparametric and prognostic approach.

Modern MRI protocols incorporate structural imaging, diffusion-weighted imaging, angiography, and advanced flow analysis techniques such as 4D flow MRI (16) and 4D MR angiography (MRA) techniques based on 3D pseudocontinuous arterial spin labeling (pCASL). Quantitative flow measurements demonstrated early and delayed reductions in shunt flow following embolization (17). These modalities provide insights into hemodynamics, venous drainage, and brain perfusion.

One key discussion is centered on the prognostic value of VOGM volume. While larger VOGMs are often associated with several venous features (including jugular bulb and superior sagittal sinus stenosis, or higher straight sinus mean diameter), absolute volume alone does not reliably predict outcomes (18). These findings support the hypothesis that VOGM size is closely linked to venous hemodynamics rather than acting as an isolated prognostic marker. Instead, a volume-to-brain ratio corrected for age at MRI may represent a promising feature in predicting survival in pre-embolization cases (19). The mediolateral diameter of the straight sinus, or falcine sinus has emerged as strong morphologic prognostic metric in VOGM (8, 10, 19). A falcine sinus width ≥7 mm was used as an eligibility criterion in the initial fetal embolization protocol (12).

While advanced MRI techniques provide valuable pathophysiological insights, standardized imaging biomarkers and longitudinal studies are still needed to clarify their role in prognostication and treatment monitoring.

## 6 Genetics and molecular insights

One of the most significant advances in recent years has been the recognition that brain AVMs are mutation-driven vascular diseases in which somatic activation of developmental pathways bypasses normal vascular stabilization. The convergence of genomic discovery, single-cell atlasing, and translational modeling provides a rationale for targeted therapies, including MEK and KRAS pathway inhibitors. This paradigm shift opens opportunities for precision medicine approaches aimed at modulating pathological angiogenesis and improving outcomes for patients with both intracranial and extracranial AVMs (20, 21).

Pathogenic variants in *RASA1* and *EPHB4* have been identified in both sporadic and familial cases, linking VOGM to broader vascular disorders such as capillary malformation–arteriovenous malformation syndrome (CM-AVM). These genes converge on Ras/MAPK signaling pathways, influencing angiogenesis and vascular remodeling. Genotype–phenotype correlations remain incomplete, and penetrance is high but variable (22). The occurrence of VOGM in monozygotic twin pairs with phenotypic discordance, with or without an identifiable germline mutation or copy number variant (CNV), has been reported. These rare observations raise the possibility that environmental and/or epigenetic factors may contribute to VOGM pathophysiology during early embryogenesis (23).

Current research is evaluating the feasibility of endoluminal biopsy during VOGM embolization for isolating endothelial somatic variants and profiling candidate second-hit mutations (24).

Despite these advances, many cases remain genetically unexplained, highlighting the need for continued research using advanced genomic and single-cell techniques.

Groundbreaking translational research on zebrafish models reveals that VOGM may result from defective signaling pathways with faulty shear-stress mechanosensing, causing failure of vascular fusion. Using zebrafish mutated for either *RASA1* or *EPHB4*, specific vascular anomalies were observed in the dorsal longitudinal vein of the zebrafish larvae's cerebral vasculature. The mutants show similar vascular anomalies and increased cardiac output as VOGM patients. Pharmacological targeting using ERK inhibitors and PI3 K activators was successful in restoring proper vessel fusion, suggesting potential pharmacological therapeutic applications (25, 26).

## 7 Risk stratification and clinical scoring systems

Accurate risk stratification is essential for guiding treatment decisions and improving outcomes. The historical Bicêtre score has long been used to assess neonatal severity and determine the timing of intervention. However, advances in clinical care have exposed its limitations. The score was developed in an era when severe neonatal presentation was frequently considered a marker of treatment futility, whereas advances in neonatal intensive care, neurointerventional techniques, and multidisciplinary management have expanded the population that can be successfully treated. Importantly, the Bicêtre score provides a predominantly clinical assessment of cardiac, respiratory, neurological, renal, and hepatic dysfunction but does not directly incorporate cerebral parenchymal injury, VOGM architecture and dynamics, or quantitative hemodynamic and perfusion parameters. Several contemporary series have therefore questioned the use of rigid Bicêtre thresholds for determining treatment versus palliation, particularly in neonates with potentially reversible systemic dysfunction (7, 8, 14).

The goal is to develop a tailored and dynamic risk-stratification system that captures the clinical complexity of modern VOGM management. Prospective multicenter validation will be necessary before such tools can be incorporated into routine decision-making.

Leveraging the ongoing academic collaboration the new system would aspire to enhance multidisciplinary decision-making, harmonize international practice, facilitate standardized research, and improve survival and long-term outcomes.

## 8 Beyond survival—clinical outcomes and long-term follow-up

Historically, outcome data for neonates and children with VOGM has focused on mortality, or overall morbidity, with relatively few studies reporting detailed neurologic outcomes. Those that do provide detailed data focus on cohorts that are now over a decade old and use a variety of measures to define outcomes. Therefore, results are difficult to compare, as they vary considerably in the assessment tools employed and the timing of follow-up. In general, “favorable outcomes” rates range from 50% in older neonatal cohorts to 70% in more recent studies, with best results in case of treatment after the neonatal period. Approximately 10% of survivors are diagnosed with epilepsy and/or autism spectrum disorder (7, 27–34).

In this perspective, the shift from pragmatic, survival-focused triage to modernized, conventional management of VOGM contextualizes advancements in care, highlighting the duty of assessing long-term quality of life and specific neurodevelopmental outcomes (35).

Although many patients achieve favorable overall outcomes, a substantial proportion still experiences long-term neurodevelopmental challenges. Specific neuropsychological and adaptive domains can be discretely affected in school-aged children, necessitating targeted surveillance and timely intervention. These may include cognitive deficits, processing speed impairment, behavioral and emotional issues. Importantly, even patients classified as having “good” outcomes may exhibit subtle but clinically significant difficulties in executive functions or mental health (28, 29).

The critical need for standardized, shared outcome measures should be emphasized. As demonstrated in other fields such as pediatric stroke (36), a unified assessment protocol enables multicenter collaboration and harmonizes follow-up across heterogeneous cohorts. To address this gap, a standardized multidisciplinary evaluation protocol should be developed, integrating neuropsychological and emotional-behavioral assessments with caregiver-reported and functional measures to enable more comprehensive and consistent evaluation of long-term outcomes.

Management of grown-up patients represents an emerging aspect in VOGM care. Preliminary evidence in adult patients showed that achievement of varix radiographic cure before the age of 18 years may improve long-term functional and neurological outcomes (37). In this perspective, pursuing early cure in patients with VOGM has the potential to offer long-term benefits and reduce the necessity for subsequent treatment.

## 9 Interventional strategies and technical advances

Advances in understanding embryology, vascular anatomy, endovascular technology, and peri-procedural management are reshaping care. Endovascular treatment remains the cornerstone of VOGM management and the meeting provided an in-depth exploration of current techniques.

The endovascular management of VOGMs can be performed through arterial, venous, or combined approaches, depending on the angioarchitecture of the lesion. The ultimate goal is durable angiographic disconnection of the pathological arteriovenous shunts, often achieved through staged embolization while preserving normal deep venous drainage (7, 39). Multiple treatment sessions are often required to achieve a satisfactory result (Figure 1).

**Figure 1:**
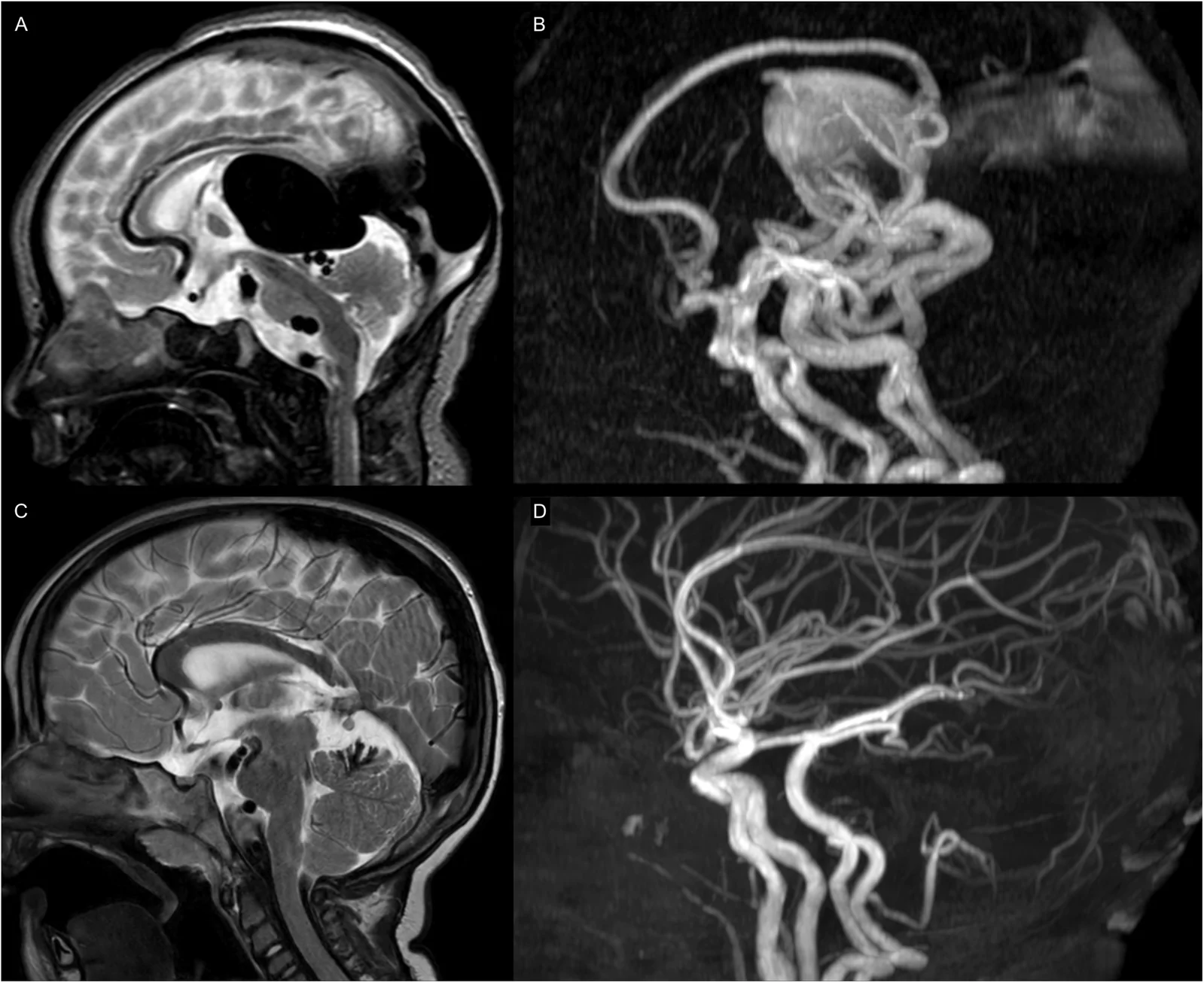
Longitudinal imaging of a patient with Vein of Galen malformation with severe neonatal presentation. **(A,B)** Neonatal brain MRI and MRA demonstrate high-flow arteriovenous shunting and marked dilatation of the median prosencephalic vein, straight sinus, and torcular Herophili. **(C,D)** Brain MRI and MRA obtained at 3 years of age following four staged endovascular embolization procedures demonstrate angiographic obliteration of the malformation with near-normal vascular anatomy.

Transarterial embolization is generally safer and preferred initially, especially for mural-type lesions with limited feeders. Transvenous embolization may be necessary for complex choroidal lesions or cases with numerous arterial feeders, although it carries higher risks due to potential compromise of critical venous drainage pathways (39). The importance of understanding venous anatomy—particularly deep venous drainage—is strongly emphasized (38). Failure to recognize critical venous connections can result in catastrophic complications, underscoring the need for meticulous pre-procedural imaging and planning (39).

Experts also reviewed the limitations of existing endovascular devices and proposed improvements. Desired innovations include shorter and more flexible catheters and microwires suitable for smaller pediatric vessels, miniaturized systems, improved liquid embolic agents with better flow control, and novel transvenous devices capable of gradually reducing venous outflow through controlled thrombosis.

Adjunctive therapies were explored. Anticoagulation following embolization may reduce cerebral venous thrombosis and suppress angiogenic responses that contribute to lesion recurrence. Experience in related pediatric cerebrovascular shunts suggests that anticoagulation may influence venous remodeling and angiogenic activity; however, its role following VOGM embolization remains investigational (40). Advanced techniques for flow control were presented as innovative methods to reduce complications. Rapid ventricular overdrive pacing is an on-demand global flow suppression technique that can be used to reduce venous escape of liquid embolic during transarterial embolization in AV shunts with angioarchitectural features prone to venous escape (41).

Jugular bulb stenosis emerged as a key determinant of outcome. By impairing cerebral venous drainage, it can worsen intracranial hypertension and reduce cerebral perfusion. Management ranges from observation and embolization of the primary lesion to angioplasty or stenting in selected severe cases (42–46). For particularly challenging patients with bilateral internal jugular vein occlusion, hybrid surgical-endovascular techniques involving direct venous sinus access have shown promising results (47).

Stereotactic radiosurgery may have a role in highly selected patients with persistent residual shunting when conventional endovascular options are limited. However, its role in VOGM remains less established than endovascular therapy and requires further evaluation.

The diagnostic and classification challenges associated with VOGM were further highlighted. It was emphasized that it is essential to move beyond the rigid dichotomy between “true” aneurysmal malformation of the vein of Galen and aneurysmal dilatation of the vein of Galen (VGAD) due to brain AVM/AVF draining into the vein of Galen. Clinical cases increasingly demonstrate that many lesions fall between these two archetypes. Rather, a spectrum exists, extending from unambiguous VOGM to unambiguous diencephalic or mesencephalic AVM/AVF, with numerous hybrid or ambiguous lesions in between. These observations underscore the limitations of classification systems and the need for individualized anatomical and physiological assessment.

## 10 The International Pediatric Stroke Study and the VOGM registry

Given the rarity and clinical heterogeneity of VOGM, large multicenter datasets are critical for advancing our understanding of its epidemiology, clinical course, treatment approaches, and long-term outcomes. The International Pediatric Stroke Study provides a global collaborative platform designed to investigate pediatric cerebrovascular diseases through standardized data collection and multinational research collaboration. Within this framework, a specific VOGM registry was established in May 2024 to collect detailed clinical, imaging, treatment, and outcome data from affected patients. This international collaborative effort seeks to define the epidemiology of VOGM, identify risk factors associated with disease progression and outcomes, and generate the evidence needed to support risk stratification and guide clinical management. By integrating multidisciplinary expertise and standardized data collection, the registry represents an important step toward optimizing care and long-term outcomes for children affected by this rare and complex vascular malformation. The operational timeframe for longitudinal data collection, along with research priorities and future clinical opportunities, was thoroughly discussed.

## 11 Family-centered care and communication of the diagnosis

Families of children diagnosed with VOGM face complex medical decisions and significant emotional challenges from the prenatal period through long-term follow-up. Comprehensive care therefore requires not only advanced medical treatment but also structured support for patients and families. A dedicated multidisciplinary VOGM team, including psychologists and case managers, provides coordinated expertise across specialties, ensuring continuity of care, and facilitating communication across the various stages of the clinical course. Educational resources, family networks, and support groups further enhance engagement and understanding. The disclosure of diagnosis to children was highlighted as a particularly sensitive issue. Best practices emphasize early, developmentally appropriate disclosure, supported by structured communication strategies and tailored educational materials (48). Evidence suggests that open and age-appropriate communication promotes better psychological adaptation, greater illness understanding, and more favorable emotional and behavioral outcomes over time (49). Together, these initiatives create an integrated family-centered care model that enhances communication, emotional support, education, and long-term engagement for children with VOGM and their families.

## 12 Conclusions

The II International Meeting on Vein of Galen Malformation in Newborns and Children provided a comprehensive overview of the current clinical and research landscape, highlighting both significant advances and ongoing challenges. Table 1 summarizes the core thematic areas addressed during the symposium.

**Table 1:** Summary of key advancements and ongoing challenges in VOGM.

Domains	Key Advancements	Future priorities
Fetal and Perinatal	Fetal MRI and ultrasound improve early detection and neurological and cardiovascular assessment. In-utero embolization may prevent postnatal hemodynamic failure.	Validation of prenatal biomarkers predicting postnatal outcomes. Assessment of safety and efficacy of fetal interventions.
Cardiovascular	Physiopathology-driven hemodynamic management improves survival and neonatal outcomes.	Development of individualized hemodynamic management strategies and consensus-based recommendations for the use of vasoactive therapies.
Neuroimaging	Comprehensive and prognostic approach. Protocols now integrate multiparametric data, like 4D flow MRI and ASL-MRA.	Development and validation of standardized neuroimaging biomarkers, clarification of the role of advanced flow-imaging techniques in post-treatment monitoring, and further investigation of whole-brain perfusion to elucidate the mechanisms underlying cerebral injury and hydrodynamic dysfunction.
Genetics	In syndromic cases, VOGM has been associated with genetic alterations involving RAS/MAPK-related vascular signaling pathways (e.g., RASA1, EPHB4). Animal models studies (e.g., zebra fish) represent pivotal field for future molecular insights.	Many cases remain genetically unexplained, warranting advanced genomic studies. Single-cell analyses and endovascular biopsy approaches may refine molecular classification and diagnosis.
Risk stratification	The Bicêtre score has limited applicability in modern times.	Development and validation of an updated risk-stratification system integrating dynamic physiological, cardiac, and neuroradiological parameters. Expansion of multicenter data collection through the IPSS and development of predictive models to improve individualized prognostication.
Outcomes	Shift from pragmatic, survival-focused triage to assessment of long-term neurodevelopmental outcomes and quality of life.	Validation of a shared neuropsychological outcome toolkit for long-term follow-up.
Interventions (INR)	Arterial, venous, or combined approaches are standard of care. Evaluation of angioarchitecture of the lesion and deep venous drainage is key in treatment planning.	Evaluation of emerging materials and techniques. Definition of the role of multimodal approaches, including hybrid procedures and stereotactic radiosurgery, in complex cases.
International Collaboration and Research	VOGM registry, as part of the International Pediatric Stroke Study, was established in 2024.	To advance understanding of disease pathophysiology and clinical trajectories, validate prognostic factors, and develop evidence-based management strategies through multicenter collaborative research.
Family Care	Multidisciplinary teams provide crucial, coordinated care. Psychological support and disclosure of the diagnosis to the child are critical in patients' life.	Establish consensus on diagnosis, family counseling, and long-term psychosocial support for patients, caregivers, and families.

ASL-MRA, arterial spin labeling magnetic resonance angiography; INR, interventional neuroradiology; MRI, magnetic resonance imaging; VOGM, Vein of Galen malformation.

Key developments include progress in prenatal diagnosis, the emergence of in-utero interventions, advances in neuroimaging and genetics, and evolving endovascular techniques. Conversely, critical gaps persist in risk stratification, long-term neurodevelopmental outcome assessment, and understanding of underlying disease mechanisms.

The meeting underscored the key role of multidisciplinary collaboration, international data sharing, and patient-centered care. By bridging diverse expertise and clinical perspectives, the symposium reinforced a collective commitment to optimizing outcomes for children with this rare and complex condition. Ultimately, these proceedings reflect a rapidly evolving field—one that is successfully moving beyond simple survival toward a comprehensive focus on long-term health, neuropsychological assessment, and quality of life.
